# Manipulating Interword and Interletter Spacing in Cursive Script: An Eye Movements Investigation of Reading Persian

**DOI:** 10.16910/jemr.14.1.6

**Published:** 2021-05-31

**Authors:** Ehab W. Hermena

**Affiliations:** Cognition and Neuroscience Research Laboratory Zayed University, Dubai, UAE

**Keywords:** Reading, eye movement, eye tracking, Persian language, Arabic script, interletter spacing, interword spacing, allography

## Abstract

Persian is an Indo-Iranian language that features a derivation of Arabic cursive script, where most letters within words are connectable to adjacent letters with ligatures. Two experiments are reported where the properties of Persian script were utilized to investigate the effects of reducing interword spacing and increasing the interletter distance (ligature) within a word. Experiment 1 revealed that decreasing interword spacing while extending interletter ligature by the same amount was detrimental to reading speed. Experiment 2 largely replicated these findings. The experiments show that providing the readers with inaccurate word boundary information is detrimental to reading rate. This was achieved by reducing the interword space that follows letters that do not connect to the next letter in Experiment 1, and replacing the interword space with ligature that connected the words in Experiment 2. In both experiments, readers were able to comprehend the text read, despite the considerable costs to reading rates in the experimental conditions.

## Introduction

Persian is an Indo-Iranian language, a subdivision of Indo-European languages (62). It is estimated to be spoken by about 110 million people worldwide (https://en.wikipedia.org/wiki/Persian_language). Modern Persian alphabet, a derivation of the Arabic script, features thirty-two letters. Similar to Arabic script, Persian script is written from right-to-left. It is also a cursive script, with the majority of the letters connected with ligatures within words. As the case with the majority of Arabic letters, most Persian letters change form depending on where they occur in a word: beginning (connected to subsequent letters), middle (connected to letters on both sides), or end, connected only to previous letters (e.g., the letter س /s/ which appears as سـ at the beginning, ـسـ in the middle, or ـس at the end of a word). When letters are connected, this connection is established by adding a flat ligature that is typically extendable (e.g., ordinary size ligatures: سـ ـسـ or ـس, compared to when each ligature is extended by a factor of three: ســـ ـــســـ or ـــس). An important implication of this is that the distance between letters within a word in this script is mostly demarcated by a horizontal black line, not white space (e.g., the trigram بسيـ /bsi/, which looks like this if the distance between the letters is increased by expanding the ligature: بــســيـــ). Only seven Persian letters do not connect to the following letter: ا, د, ذ, ر, ز, ژ, and و. Thus, whereas there is always white space to demarcate word boundaries in Persian, that is, interword spacing, the distance between the letters in a word, or interletter spacing, may be composed of a combination of ligatures if the letters are connectable, and white spaces (e.g., the word بسيار features white space between the last two letters, ـار, but the rest of the letters before are ‘separated’ by ligature. Thus, the allographic nature of Persian letters and cursive script, similar to Arabic, makes it an interesting script to use in studying eye movement control during reading. In the reported experiments the effects of manipulating word spacing and the distance between letters were investigated in reading Persian sentences.

Interword spacing, or the space between words in text, has been shown to play an important role in facilitating reading. This space allows readers to segment the text and perform word identification. Previous findings showed that removing or filling this space resulted in delaying word identification in sentence reading and thus slowing reading rate considerably (estimated 30-50% decrement, e.g., 12, 29, 32, 43, 44, 53, 54, 61, 64). The interword spacing also allows readers to benefit from the important information conveyed by each word’s first and last letters that play an important role in word identification (10, 16, 21, 22). It is not surprising thus that moderate increases in interword spacing was found to facilitate reading in numerous studies (11, 2, 30, 57). Increasing interword spacing was suggested to aid in text segmentation and word identification, and this improves reading performance given that words are the main units of linguistic processing during reading (e.g., 57).

Furthermore, when considering the readers’ eye movements during text reading, the presence of space between words provides the necessary spatial frequency information needed in saccade targeting so that fixations may land on optimal spot for uptake of visual information (i.e., the preferred viewing location, PVL, 39). Removing these spaces results in significant changes to the saccadic targeting system, with readers’ fixations landing in suboptimal locations, closer to word beginning, as well as shortening saccade amplitude (e.g., 30, 32, 40, 53, 64).

Beyond the superficial visual processing of text, removing the space between words was found to disrupt the core linguistic processes of word identification. Several studies showed evidence of this by including in the sentences target words of high and low frequency. Word frequency effects are typically considered an indicator of the time course of lexical processing, with higher frequency words being identified earlier (i.e., faster) than lower frequency words (40, 49). Indeed, several investigations showed that in the absence of interword spacing, frequency effects are amplified (e.g., 30, 32, 40, 53, 54). Furthermore, and more indicative of the disruption to word identification when interword spacing was removed, analyses of distributions of fixation times showed that the onset of word frequency effects was delayed, relative to when the interword spaces were preserved (53, 54). There are languages that do not feature interword spacing (e.g., Chinese and Thai), however, segmentation and word boundary identification is of equal importance in these languages (e.g., 1, 18, 19, 24, 63).

Interletter spacing, or the space between the letters in a word also plays a role in word identification, albeit a role that requires further clarification. Reducing this space and making letters appear closer to each other increases visual crowding, or the phenomenon that a middle letter would be slower to identify if flanked by two close outer letters (e.g., 5, 6, 9). Increasing interletter spacing and reducing crowding results in increased perception of letter size (55). Subtle increases in this space were reported to facilitate lexical decision (35, 34). In sentence reading, subtle increases in letter spacing (+0.5 and +1.0 pixel conditions) was associated with reduction in average fixation duration, but an increase in the total number of fixations, relative to normal, unaltered, letter spacing, with the latter condition resulting in the shortest total sentence reading time (57, Experiment 1). On the other hand, Slattery and Rayner found that decreasing letter spacing by 0.5 pixel resulted in higher average fixation durations, increased number of fixations, and longer total sentence reading time relative to the unaltered interletter spacing condition. Interestingly, Slattery and Rayner found that manipulating interletter spacing had no significant effect on the rate of target word skipping (the word is not fixated at all during first pass reading). Similarly, this letter spacing manipulation had no significant effect on the location of the initial fixation this target word received, with these first fixations always landing at the optimal position between the word beginning and center. Slattery and Rayner concluded that the saccade targeting system rapidly adjusts to the spacing manipulation and continues to optimally serve the process of reading.

Other investigations revealed that any benefits of increasing interletter spacing asymptote, and even reverse, after a certain point in word identification tasks (e.g., 8, 28, 30, 31, 51, 58), with sizable disruptions reported when interletter space extends beyond 2-3 character spaces. Clearly, as interletter spacing increases, more characters are pushed out of foveal vision, and less information becomes available parafoveally as more and more characters are pushed further from fixation location. This has a detrimental effect on sentence reading that depends on the availability of foveal (fixated) and parafoveal (upcoming) information (see 43, 41, 52). Some investigations revealed that readers compensate for increased interletter spacing by making more fixations, of shorter duration, relative to when reading normally-spaced texts, thus producing largely comparable overall sentence reading times in both conditions (e.g., 11, 27, 33, 40). Notable inconsistencies of the reported results from interletter spacing manipulations were attributed to the different fonts used in the different investigations, given the natural, and sizable, differences in letter spacing in different fonts. For instance, monospaced fonts that render all characters, including spaces of equal size (e.g., Courier New), feature larger letter spacing than proportional fonts that allow for character spaces to vary naturally (e.g., Times New Roman, where the characters I naturally occupy narrower space than the character W; see relevant discussions in 17 for fonts used in Arabic script; 35, 58, 57, 56, 60).

The two experiments reported here investigate the effects of reducing interword spacing, and increasing the distance (ligature) between Persian letters within words on eye movement behavior, and on sentence comprehension when reading Persian sentences. These experiments are part of an on-going series of investigations at our labs of interword and interletter spacing in Arabic and Persian as examples of cursive scripts.

## Experiment 1

**Figure 1.**
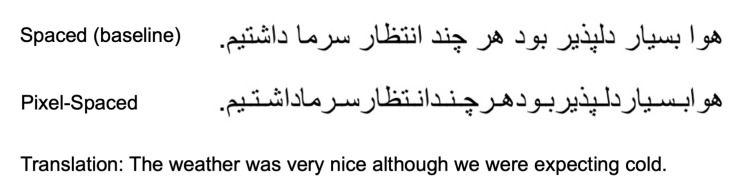
Sample of the stimuli sentences used in Experiment 1. The sample shows both the Spaced (baseline) and the Pixel-Spaced conditions.

In this experiment the readers were presented with two conditions: A baseline condition with no manipulation of word or letter spacing or distance; and an experimental condition where interword spacing was reduced such that the words were almost touching (what will be referred to as the Pixel-Spaced condition, see Figure 1 for an example). The reduction of interword spacing in the Pixel-Spaced condition was accompanied by an interletter compensation such that the space before each word was added to the word itself in the form of extended ligature, thus increasing the distance between letters within the word. For example, printed with normal spacing, the two words هوا بسيار (= the weather was very…) can appear like this هوابـسـيار with the space between them removed and added to the second word بسيار, in effect pushing the letters بـ ـسـ and ـيـ away from each other by the same amount of space that was removed from between the words (see also Figure 1). In a sense, this manipulation is the opposite of one of the manipulations of Slattery and Rayner (57). In their second experiment, Slattery and Rayner found the combination of reducing interletter spacing, and increasing interword spacing resulted in facilitation in sentence reading (reduced fixation durations). As such, the opposite effects (i.e., processing cost) may be expected in this condition, given that the Pixel-Spaced manipulation increased the distance (ligature) between the word’s letters and reduced interword spacing.

However, there is an alternative scenario. Namely, increasing the distance between the letters within the words in the Pixel-Spaced condition may equate to reducing crowding and lateral inhibition, and thus some benefit maybe observed. Importantly, the stimuli sentences in this experiment featured words that ended with letters that cannot be connected to the following letter (i.e., one of the letters ا, د, ذ, ر, ز, ژ, and و), that is, letters that naturally insert white space between letter strings and thus may effectively serve as word-end markers. One of the aims of this experiment is thus to determine whether readers use these letters as word-boundary markers. If the presence of these letters at the end of the word, combined with increasing the distance (ligature) between the letters within words facilitates word identification, it would be possible to offset, at least to some extent, the costs expected for dramatically decreasing interword spacing in the Pixel-Spaced condition.

## Methods

### Participants

The same set of participants took part in both experiments. Twenty-eight participants (six men) took part in the experiments. All participants were native Persian speakers living in the UAE, and all indicated that they regularly read Persian, on daily or weekly basis. The participants’ mean age was 35.7 years (SD = 8.7, range = 18 – 50). All participants had normal or corrected to normal vision as determined by the Bailey-Lovie chart (2).

### Materials

Forty simple Persian sentences were used as stimuli, and were presented to the participants in either normally spaced (baseline condition), or with the space between the words (interword spacing) reduced significantly (the Pixel-Spaced condition). An example of the sentence used is available in Figure 1. With the exception of the last word in each sentence, all words ended with letters that cannot be connected to the following letter. The sentences comprised, on average, 10.4 words (SD = 2.1, range = 6 – 15 words). This was about 48.8 characters per sentence (including interword spaces in the Spaced condition, or the within-word ligatures that replaced this space in the Pixel-Spaced condition, SD = 8.4, range = 30 – 64 characters). The amount of physical space the sentences occupied in the Spaced and Pixel-Spaced conditions was thus identical. The sentences were all rendered in Arial font size 14. Arial is a proportional font that allows characters to naturally vary in the amount of physical space they occupy. It is a widely-known and used font in Persian print. Additional four sentences of similar complexity and length were used as practice items for the participants.

*Stimuli norming*. For assessing the grammaticality and correctness of structure of all stimuli sentences, in both experiments, additional 5 native readers of Persian were asked to rate the sentences on these variables on a 1 – 5 scale (1 = poor grammar / structure, 5 = perfect grammar / structure), thus providing 5 ratings per sentence. Those 5 participants did not take part in the eye tracking procedure. All sentences for both experiments were rated as grammatically sound, with average rating of 4.5 (SD = 0.2, range = 3 – 5).

### Apparatus

A tower-mounted EyeLink 1000+ eye tracker was used to sample readers’ eye movements during reading. Viewing was binocular, but eye movements were recorded from the right eye only. The eye tracker sampling rate was set to 1000Hz. The eye tracker was interfaced with a Silverstone computer, and with a 24-inch BenQ monitor. Monitor resolution was set at 1920 × 1080 pixels, with the maximum vertical refresh rate (144Hz). The participants leaned on a headrest to minimize head movements. The sentences were displayed as a single line, in black on a white background. The participants viewed the screen from 78 cm, and at this distance, on average, 4.3 characters equaled 1° of visual angle.

### Design

The spacing manipulation was the within-participants independent variable. The order of sentence presentation was randomized, and the presentation of the two spacing conditions was counterbalanced such that each participant saw each sentence only once, in either the Spaced (baseline) or the Pixel-Spaced conditions.

### Procedure

The study was approved by the university’s ethics review board. At the beginning of the testing session, the participants were given the consent form package (including information sheet). Consenting participants took part in a vision acuity test before the start of the eye tracking procedure.

The eye tracker was calibrated using a horizontal 3-point calibration at the beginning of the experiment, and the calibration was validated. Calibration accuracy was always ≤ 0.25°, otherwise calibration and validation were repeated. Prior to the onset of the target sentence, a circular fixation target (diameter = 1°) appeared on the screen in the location of the first character of the sentence. When the tracker registered a stable fixation on the circle, the sentence was displayed.

The participants were told to read silently and press a button on the button box when finished reading each sentence. Additionally, they would be required to use the button box to provide a yes/no answer to the comprehension questions that followed around 40% of the sentences. Before being exposed to the experimental sentences, the participants read 4 practice sentences (also followed by yes/no questions) to become acquainted with the procedure.

In total, the participants read 104 sentences (4 practice sentences + 40 sentences in Experiment 1 + 60 sentences in Experiment 2). The participants were allowed to take breaks followed by re-calibration of the tracker. The testing session lasted around 45-50 minutes, depending on how many breaks a participant took.

## Results and Discussion

Global eye movement measures that index sentence processing are reported. These are: the average duration of fixations made during sentence reading, average number of fixations made, total sentence reading time (from the onset of the sentence until the participant pressed the button to change the display), and average amplitude (length) of saccades made during sentence reading (reported in visual angle). In addition, the average sentence comprehension score is also reported as an indicator of whether readers’ comprehension performance was affected by the spacing manipulation. Table 1 shows the descriptive statistics for these dependent measures for both spacing conditions.

**Table 1.** Descriptive Statistics for the Dependent Measures in Experiment 1.

	Spaced	Pixel-Spaced
Average Fixation Duration	246 (42.53)	265 (40.77)
Average Fixation Count	12.9 (5.37)	16.4 (8.49)
Total Reading Time	3687 (1628.95)	5002 (2931.01)
Average Saccade Amplitude	1.6 (0.50)	1.5 (0.65)
Sentence Comprehension	96% (0.21)	95% (0.22)

Note. Average fixation duration and total reading time are reported in milliseconds. Saccade amplitude is reported in degrees of visual angle. Standard deviations are between parentheses.

The lme4 package (version 1.1-26, 4) was used within the R environment for statistical computing (38) to analyze all dependent measures by fitting generalized linear mixed-effects models (GLMMs), with Gamma-distribution assumed for the fixation duration measures (Average Fixation Duration and Total Reading Time). Using GLMMs to analyze raw positively-skewed response times, including fixation durations, maintains the transparency of the reported analyses while satisfying the necessary normality assumptions, without the need to transform data (25). For the sentence comprehension measure, logistic GLMM was used to account for the binary nature of this variable. In these models the spacing condition was the fixed variable, and subjects and items were the random variables. Models with maximal random structure were always the start point (3). Model trimming was carried out when failure to converge occurred, or when singular boundaries (suggesting overparameterization) were identified. All findings reported here are from successfully converging models. For each measure the beta values (b), standard error (SE), t statistic, and the associated *p* value are reported in Table 2.

**Table 2.** Linear Mixed Models Outputs for the Measures Reported in Experiment 1.

	*B*	*SE*	*t*	*p*
	Average Fixation Duration			
(Intercept)	253.33	9.16	27.66	< .0001
Spaced vs. Pixel-Spaced	17.69	4.55	3.89	.0001
	Average Fixation Count			
(Intercept)	12.93	0.73	17.70	< .0001
Spaced vs. Pixel-Spaced	3.41	0.55	6.23	< .0001
	Total Reading Time			
(Intercept)	4097.36	16.65	246.00	< .0001
Spaced vs. Pixel-Spaced	1485.12	14.00	106.10	< .0001
	Average Saccade Amplitude			
(Intercept)	1.63	0.07	23.21	< .0001
Spaced vs. Pixel-Spaced	-0.16	0.04	-4.09	.0004
	Sentence Comprehension			
(Intercept)	3.68	0.46	7.94	< .0001
Spaced vs. Pixel-Spaced	0.21	0.62	0.35	.7290

As Tables 1 and 2 show, reducing the interword spacing and increasing the interletter distance in the Pixel-Spaced condition resulted in significant increases in average fixation duration, average fixation count, and in total sentence reading time. By contrast, saccade amplitude was significantly reduced in the Pixel-Spaced condition. Readers comprehension scores, however, indicated that they were still able to successfully comprehend the sentences they were reading, in both conditions, albeit with longer sentence reading time in the Pixel-Spaced condition.

The obtained results replicate previous findings where reducing interword spacing (the space between words) was detrimental to reading speed (see literature review above). Importantly, the results suggest that there was no clear benefit from increasing the distance (ligature) between the letters within a word. Furthermore, if readers used the Persian letters that do not connect to the next letter as word-boundary markers, the results show that dramatically reducing the white space that follows these letters results in similar reduction to reading speed as is the case in other non-cursive scripts. In other words, for Persian readers, word segmentation is more dependent on having normal-sized white space between the words, rather than relying on any cues from the letters that do not connect to the next letter. This is perhaps not surprising since such letters do regularly occur in the middle of words, as well as at word ends. These findings will be discussed in more detail in the General Discussion.

## Experiment 2

**Figure 2.**
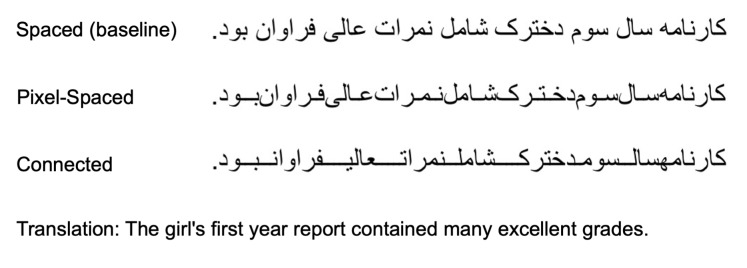
Sample of the stimuli sentences used in Experiment 2. The sample shows the Spaced (baseline), the Pixel-Spaced, and Connected conditions.

This experiment aimed to replicate and expand on the findings of Experiment 1. The main difference between the two experiments was that in the current experiment the sentences used words that ended with letters that can be connected to the next letter. Experiment 2 thus featured Spaced (baseline) and Pixel-Spaced conditions, same as in Experiment 1. In addition, there was a third condition where the space between the words was replaced by ligature that connected the words. This condition will be referred to as the Connected condition. As opposed to extending the interletter space (ligature) within words in the Pixel-Spaced condition, in the Connected condition the interword spacing was completely replaced by between-word ligatures, without affecting the interletter distance within words (see Figure 2).

Completely filling the white interword space in previous investigations resulted in significant disruption to reading, as discussed above (e.g., 40 replacing the spaces with the character x; 53 replacing the space with random numbers; etc.). However, none of these investigations reported a manipulation that involved cursive, connected-by-ligatures, text. Additionally, none of these investigations, also changed the appearance of the final and first letters of the words being connected as is the case when replacing the space between Persian words with connecting ligatures (see Figure 2). Importantly, connecting words by ligatures can be considered as a very strong manipulation that would compromise word segmentation cues in a way not possible in non-cursive scripts (e.g., European languages). In addition to significantly altering the appearance of the words’ first and last letters, this manipulation provides inaccurate information about word boundaries, which can be expected to slow readers down. Thus, in addition to expecting to replicate the disruption to reading in the Pixel-Spaced condition, it is plausible to expect even greater disruption to reading in the Connected condition given the loss of the white interword spacing, and the profound change to the first and last letters of the words when connected. As detailed above, these letters play an important role in word identification.

## Methods

The participants, apparatus, and procedure in this experiment were identical to Experiment 1.

### Stimuli

Sixty simple Persian sentences were used as stimuli, and were presented to the participants in the Spaced (baseline) condition, the Pixel-Spaced condition, or the Connected condition, that is, with the interword spacing replaced by ligatures, as explained above. An example of the sentences used are available in Figure 2. The sentences comprised, on average, 8.9 words (SD = 1.5, range = 7 – 13 words). This was about, on average, 47.8 characters per sentence (including interword spaces in the Spaced condition, or the within-word ligatures that replaced this space in the Pixel-Spaced condition, or the between-word ligatures that replaced this space in the Connected condition, SD = 6.5, range = 35 – 62 characters). The amount of physical space the sentences occupied in all three conditions was thus identical. The sentences were also rendered in Arial font size 14.

### Design

The spacing manipulation was the within-participants independent variable. The order of sentence presentation was randomized, and the presentation of the three spacing conditions was counterbalanced such that each participant saw each sentence only once, in either the Spaced (baseline), the Pixel-Spaced, or Connected conditions.

## Results and Discussion

The same dependent measures reported in Experiment 1 are reported in Experiment 2. Table 3 provides the descriptive statistics for these dependent measures for all three spacing conditions.

**Table 3.** Descriptive Statistics for the Dependent Measures in Experiment 2.

	Spaced	Pixel-Spaced	Connected
Average Fixation Duration	236 (38.67)	238 (35.02)	311 (50.85)
Average Fixation Count	12.8 (5.35)	13.5 (5.12)	36.1 (22.49)
Total Reading Time	3547 (1561.82)	3742 (1528.79)	12937 (9218.88)
Average Saccade Amplitude	1.9 (0.65)	1.8 (0.60)	1.2 (0.65)
Sentence Comprehension	92% (0.27)	91% (0.29)	85% (0.36)

Note. Average fixation duration and total reading time are reported in milliseconds. Saccade amplitude is reported in degrees of visual angle. Standard deviations are between parentheses.

The same inferential analyses described in Experiment 1 were used in Experiment 2, using GLMMs within the R environment. Two contrast matrices were pre-specified for the GLMM models. In the first matrix, the Spaced condition was treated as the baseline against which the Pixel-Spaced and Connected conditions were contrasted. In the second matrix, Pixel-Spaced and Connected conditions were contrasted. The full output of these analyses is reported in Table 4.

*Spaced vs. Pixel-Spaced Contrast*. As Tables 3 and 4 show, the results obtained largely replicate those reported in Experiment 1, albeit with a reduced magnitude of effects for the Pixel-Spaced condition in Experiment 2. The small (and non-significant) increases in average fixation duration and average fixation count measures in the Pixel-Spaced condition translated into a significant increase in total sentence reading time in this condition. Also as seen in Experiment 1, saccade amplitude was significantly reduced in the Pixel-Spaced condition. And there was no significant difference in reading comprehension between the two conditions.

**Table 4.** Linear Mixed Models Outputs for the Measures Reported in Experiment 2.

	*B*	*SE*	*t*	*p*
	Average Fixation Duration			
(Intercept)	242.46	7.81	31.05	< .0001
Spaced vs. Pixel-Spaced	1.34	2.66	0.50	.6150
Spaced vs. Connected	76.69	5.52	13.90	< .0001
Pixel-Spaced vs. Connected	-34.17	2.77	-12.35	< .0001
	Average Fixation Count			
(Intercept)	12.80	0.87	14.64	< .0001
Spaced vs. Pixel-Spaced	0.70	0.64	1.09	.2760
Spaced vs. Connected	23.26	2.01	11.55	< .0001
Pixel-Spaced vs. Connected	-11.29	1.00	-11.28	< .0001
	Total Reading Time			
(Intercept)	3963.13	11.77	336.84	< .0001
Spaced vs. Pixel-Spaced	200.66	12.15	16.52	< .0001
Spaced vs. Connected	8437.33	11.37	741.78	< .0001
Pixel-Spaced vs. Connected	-3235.15	9.61	-336.50	< .0001
	Average Saccade Amplitude			
(Intercept)	1.88	0.08	22.95	< .0001
Spaced vs. Pixel-Spaced	-0.11	0.03	-3.24	.0032
Spaced vs. Connected	-0.72	0.07	-10.87	< .0001
Pixel-Spaced vs. Connected	0.30	0.03	10.58	< .0001
	Sentence Comprehension			
(Intercept)	-25.35	7.88	-3.22	.0013
Spaced vs. Pixel-Spaced	9.89	8.08	1.22	.2209
Spaced vs. Connected	14.34	8.05	1.78	.0750
Pixel-Spaced vs. Connected	-0.89	1.31	-0.68	.4950

*Spaced vs. Connected Contrast*. There were sizable and significant costs for filling the interword spacing with ligature in the Connected condition whereby significant increases in average fixation duration, average fixation count, and total sentence reading time were observed. Saccade amplitude was also significantly shorter in the Connected condition. The difference between the two conditions in sentence comprehension was however not statistically reliable, with readers still scoring 85% accuracy on the Connected condition.

*Pixel-Spaced vs. Connected Contrast*. Once again, there were sizable and significant costs for filling the interword spacing with ligature in the Connected condition relative to the Pixel-Spaced condition. Significant increases in average fixation duration, average fixation count, and total sentence reading time were observed. Saccade amplitude was also significantly shorter in the Connected condition. And the two conditions did not differ significantly in sentence comprehension.

In addition to largely replicating the sentence reading disruption observed in the Pixel-Spaced condition in Experiment 1, the results clearly show a massive disruption to reading in the Connected condition. Replacing interword spacing with ligature, and connecting the words thus altering the form of the first and last letters proved to be detrimental to the speed of reading, as predicted. Participants’ performance on reading comprehension was, however, still comparable in all conditions. These findings will be discussed in more detail below in the General Discussion.

## General Discussion

The reported experiments aimed to use the properties of Persian script to explore how eye movement behavior and reading performance were affected by: (a) reducing interword (between words) spacing, while increasing the interletter distance (ligature) within words (the Pixel-Spaced conditions in both experiments), and (b) replacing the interword space with connecting ligature (the Connected condition in Experiment 2). In addition, Experiment 1 aimed to explore whether readers use Persian letters that do not connect to the following letter as word boundary markers.

The results obtained were unequivocal. With regards to reading rate, the severe reduction in interword spacing combined with increasing the interletter distance (ligature) within words in the Pixel-Spaced condition resulted in significant reading disruption in Experiment 1. This was largely replicated in Experiment 2. Amongst the mechanisms that can account for the reported results is that decreasing the interword white space in the Pixel-Spaced conditions may have resulted in the words’ first and last letters suffering increased crowding effects and lateral masking (e.g., 5, 6, 59). The significant disruption to reading rate observed in the Connected condition indicates that not only the distance between characters is important, but also preserving the physical allographic form of these characters is also important for successful text segmentation and word identification. It will be recalled that connecting the first and last letters by ligature resulted in altering the physical forms of these letters.

**Figure 3.**
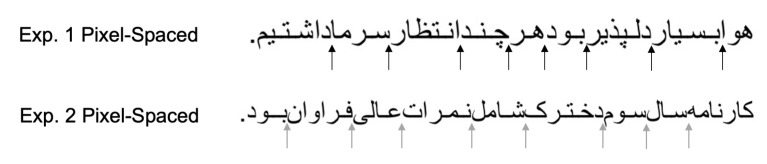
The Pixel-Spaced conditions in both experiments. The black arrows indicate where reducing the interword space, following the letters that do not connect to the next letter may have resulted in inaccurate word boundary cues in Experiment 1. The grey arrows indicate where the words’ final letters in Experiment 2 could be connected to the next letter, but were not, thus perhaps providing word segmentation cues to the readers.

With regards to the question whether readers use letters that do not connect to the next letter as word boundary markers, contrasting the results from both experiments was informative. Specifically, the results showed that reducing the white space that followed these letters (Experiment 1) yielded more sizable effects (e.g., the measures of average fixation duration and count, and total reading time) than reducing the space that followed the other letters that can be connected to the next letter (Experiment 2). One explanation may have to do with the fact that the letters that do not connect to the next letter regularly fall in the middle of words, and when they do, they are followed by a very small white space that separates them from the next letter within the word. As such, reducing the space that followed these letters in Experiment 1 potentially provided inaccurate word segmentation information to the readers (i.e., making them look like interletter and not interword spaces). Reading was thus significantly disrupted in the Pixel-Spaced condition in Experiment 1. By contrast, in Experiment 2, the fact that letters that can be connected to the next letter by ligatures remained unconnected in the Pixel-Spaced condition may have provided a valuable word boundary cue that somewhat attenuated the effect of reducing the interword space (see illustration in Figure 3). We can thus conclude that preserving or violating word segmentation cues is more important for reading than particular letter properties, such as the possibility of connecting to the next letter, per se. This is in line with findings that showed that text spacing that violates word boundaries was found to be particularly detrimental to reading rate (e.g., 1, see also 14, 15, 29, 36, 40, 53, 58).

A sizable disruption to reading was observed when the interword spaces were replaced by word-connecting ligature that also altered the form of the words’ first and final letters. As outlined above, connecting words by ligatures is a strong manipulation that compromise word segmentation cues in a way not possible in non-cursive scripts (e.g., European languages). These results replicate the findings discussed above with regards to the costs of reducing interword spacing (e.g., 12, 29, 32, 40, 44, etc.), and the importance of the first and last letters in word identification (e.g., 10, 16, 21, 22). The reported results also add further support to the suggestion that text segmentation and word identification are vital for smooth reading (e.g., 57).

Furthermore, in the Connected condition (Exp. 2), reading may have been disrupted because unsegmented text is an unfamiliar visual format for Persian readers. Bai et al. (1) suggested that unfamiliar visual text format may disrupt reading and result in longer reading times relative to a more familiar format. However, as Sheridan et al. (53) pointed out, visual familiarity cannot solely account for the observed disruption to reading rate. Indeed, in English, with its relative less complex visual characteristics (e.g., letters do not change shape depending on their location in the word, save some instances of initial capitalization), lengthy training and familiarization of participants to read unspaced texts did not result in reading facilitation (e.g., 26). Future investigations should further explore the psychological reality of visual familiarity in allographic scripts (e.g., Arabic and Persian), relative to the scripts of European languages.

Thus far the main focus was on the findings concerning how reading rate was affected by the reported experimental manipulations. The reported sentence comprehension scores in both experiments, even in the Connected condition in Experiment 2, replicated previous findings that readers are still able to comprehend unspaced text (e.g., 14, 15), albeit with the sizable decreases in reading rate and efficiency (e.g., 43). It is plausible to suggest that whereas the Pixel-Spaced condition posed significant difficulty, the difficulty readers encountered in reading the Connected condition in Experiment 2 makes the obtained comprehension scores more akin to solving a visual puzzle rather than natural sentence reading. The increases in readers’ average fixation duration, fixation counts, and total reading time suggest that the eye movement behavior was guided by the attempts to segment the text, identify words (and test hypotheses about where this should be done), to facilitate the processes of extracting meaning from the visual stimuli. That readers were able to obtain such high comprehension scores indicates the resilience of the linguistic processing system, and its role in guiding eye movements.

Finally, the obtained results may be considered to lend further support to models of eye movements control that postulates serial processing and word identification in reading (e.g., the E-Z Reader model: 37, 42, 45, 47, 48, see also 46), rather than models that postulate distributed attentional grade and parallel processing of multiple words (e.g., the SWIFT model: 13, 23, 50). Specifically, in the reported experiments, disrupting word identification by increasing the interletter distance (ligature), and bringing the upcoming word closer by reducing the interword spacing (the Pixel-Spaced conditions) did not result in any facilitation to reading, rather, the opposite. However, this may not be a completely fair assumption since bringing the parafoveal word closer was achieved by reducing the interword spacing, and thus dramatically reducing the ability to segment words. Presumably, both models would predict some sort of cost associated with this. For interletter spacing, both E-Z Reader and SWIFT models would predict costs for increasing interletter spacing, given that increasing this space would lead to placing the letters further from the point of ﬁxation (e.g., see 7, 31 for discussion of interletter spacing and crowding effects). As neither model has yet simulated the effects of interword or interletter spacing manipulations, making model-derived predictions about the effects of such manipulations is not possible (e.g., 33, also 46). Further modeling activity is thus necessary to obtain further clarity.

Future investigations should further utilize the properties of cursive scripts (e.g., Arabic and Persian) to formally expand and update current theories and models to accommodate the characteristics of non-European scripts. In this regard, developing corpuses that provide letter positional probabilities (particularly the probability of letters occurring at word beginning or end, see e.g., 65) can further elucidate the extent to which readers may use such properties and rely on certain letters (more than others) as markers of word boundaries.

### Ethics and Conflict of Interest

The author declares that the contents of the article are in agreement with the ethics described in http://biblio.unibe.ch/portale/elibrary/BOP/jemr/ethics.html and that there is no conflict of interest regarding the publication of this paper.

### Acknowledgements

Many thanks to Hajar Amani for her help in planning the experiments and data collection.

Also many thanks to anonymous reviewer #1 and to Erik D. Reichle (reviewer #2) for their very helpful comments on the first draft of this article.
